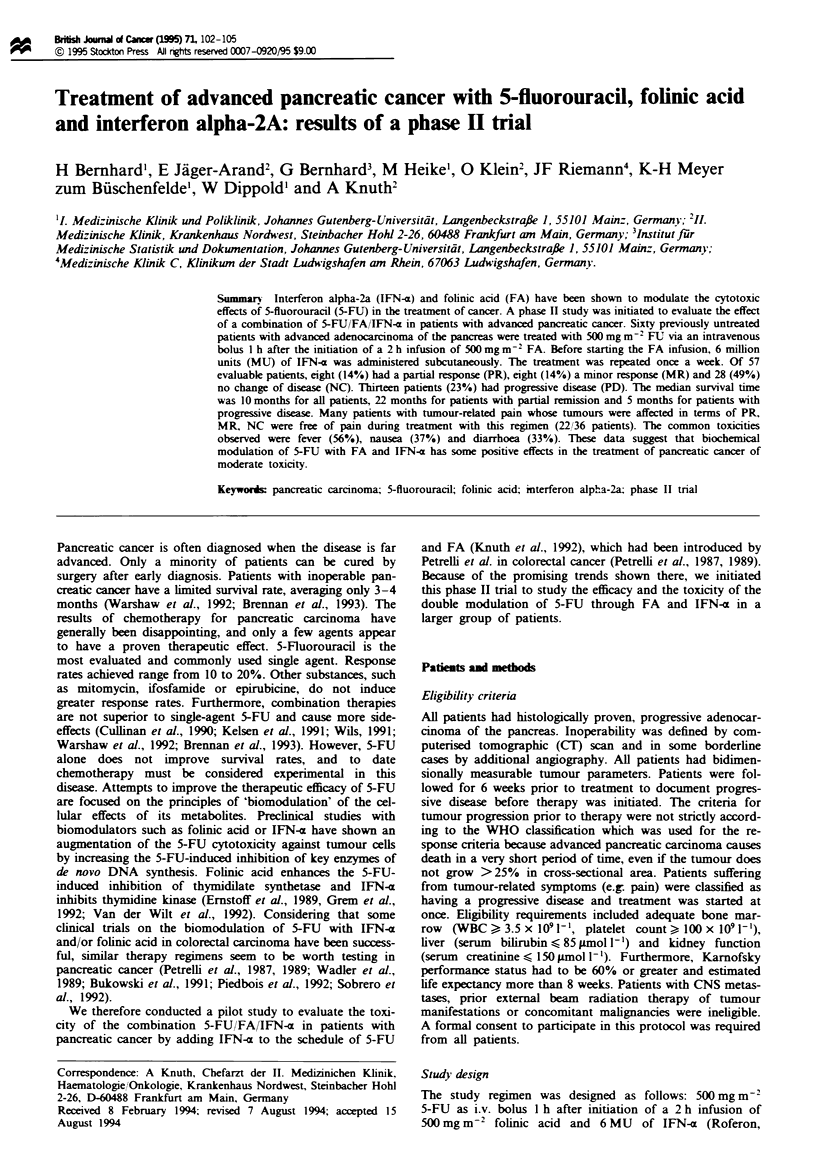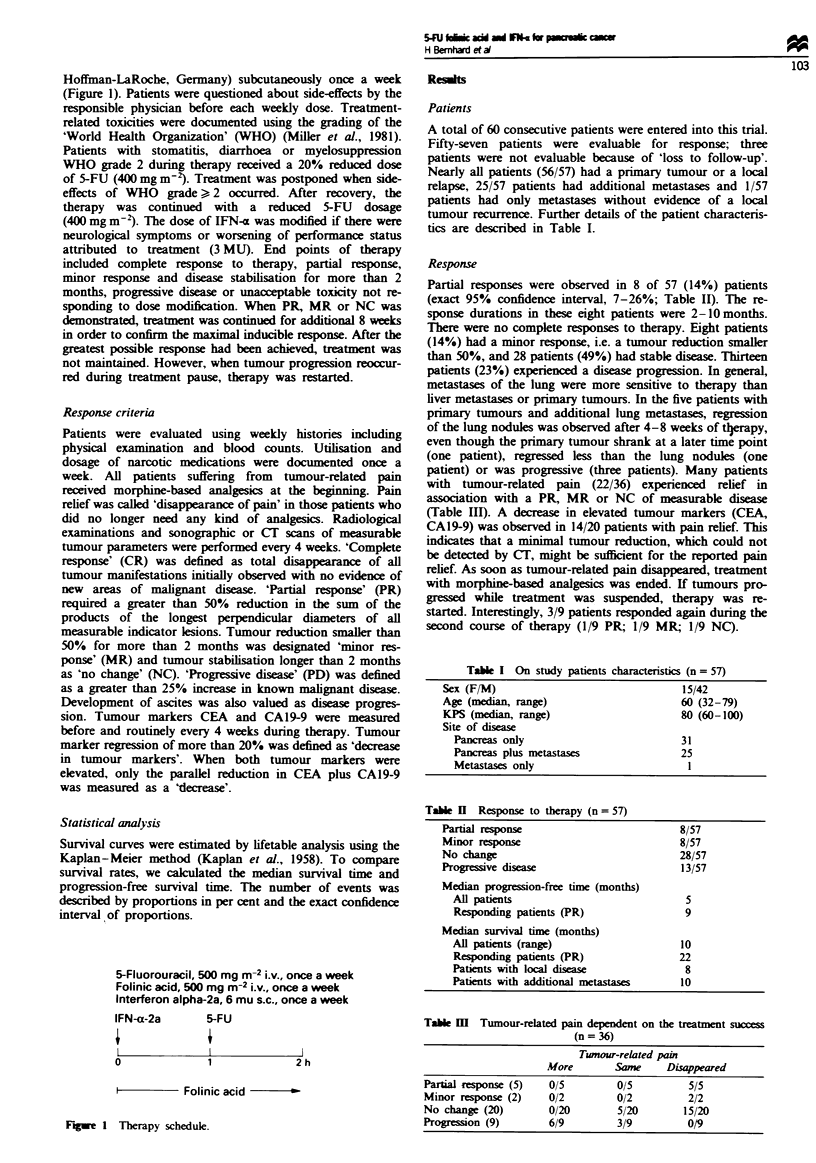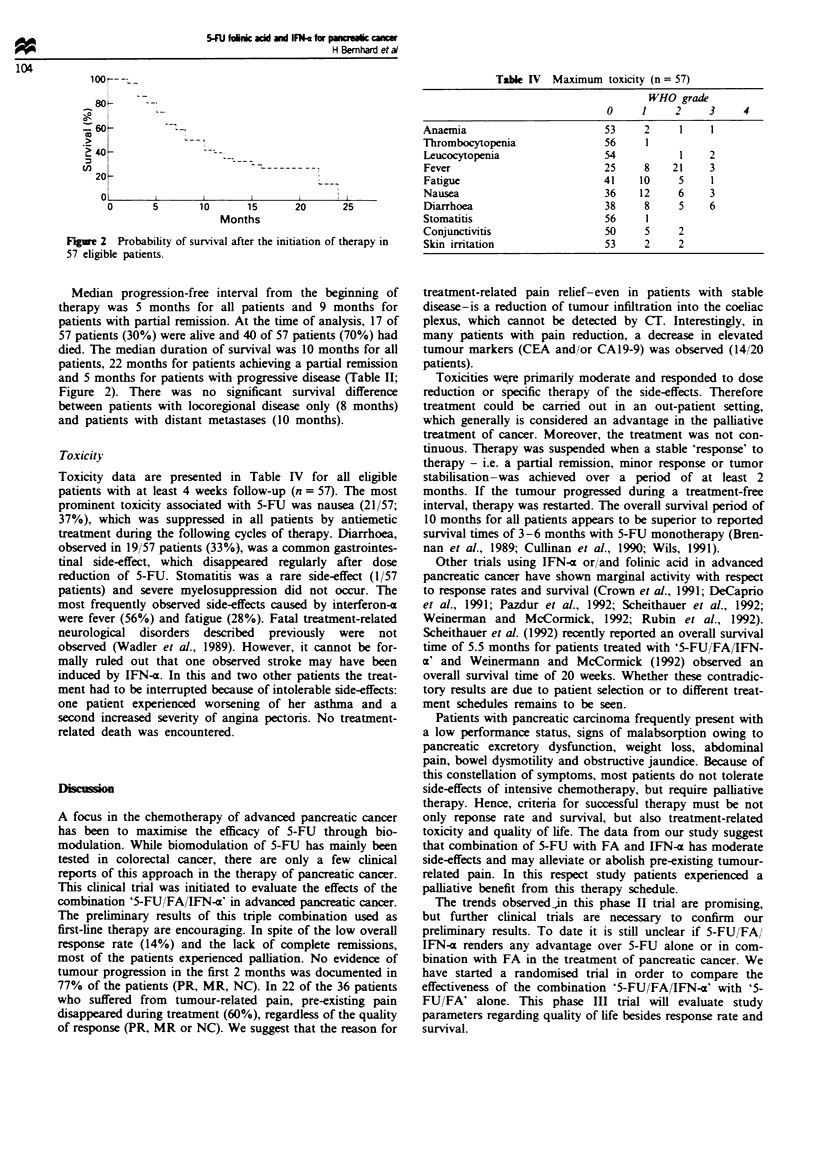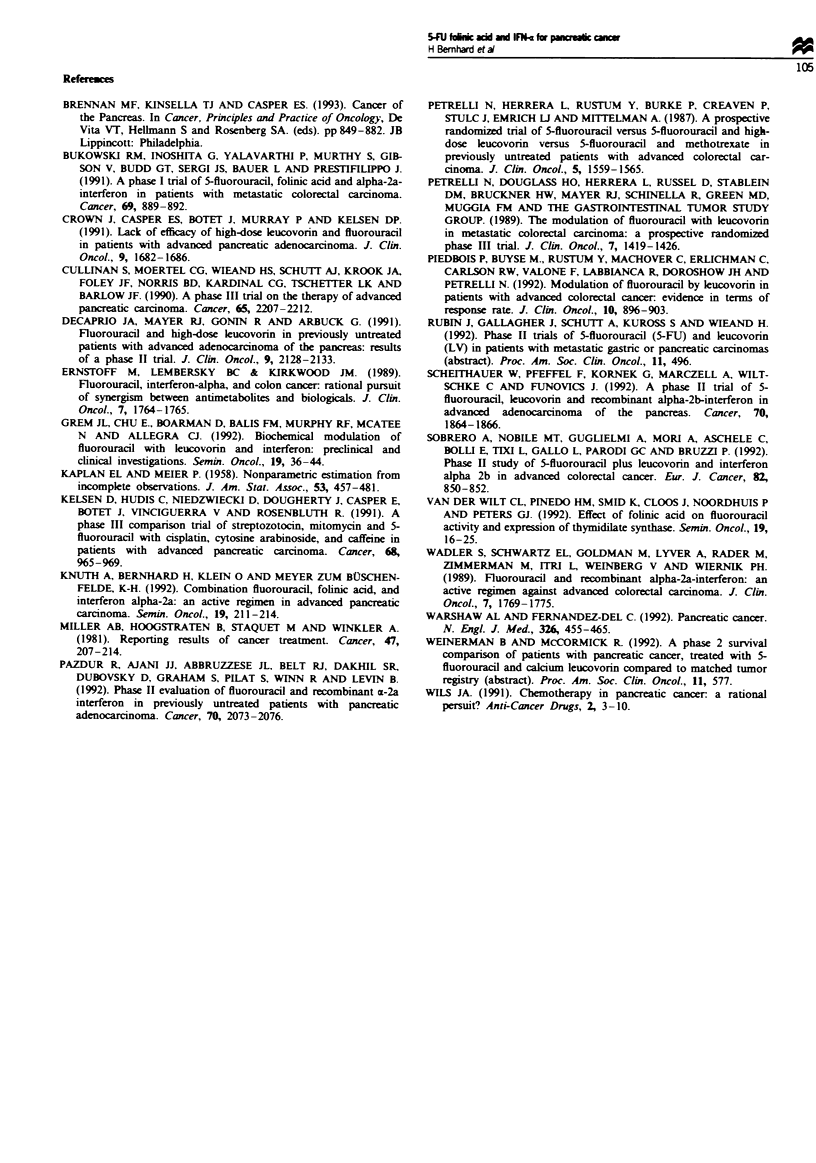# Treatment of advanced pancreatic cancer with 5-fluorouracil, folinic acid and interferon alpha-2A: results of a phase II trial.

**DOI:** 10.1038/bjc.1995.20

**Published:** 1995-01

**Authors:** H. Bernhard, E. Jäger-Arand, G. Bernhard, M. Heike, O. Klein, J. F. Riemann, K. H. Meyer zum Büschenfelde, W. Dippold, A. Knuth

**Affiliations:** II. Medizinische Klinik und Poliklinik, Johannes Gutenberg-Universität, Mainz, Germany.

## Abstract

Interferon alpha-2a (IFN-alpha) and folinic acid (FA) have been shown to modulate the cytotoxic effects of 5-fluorouracil (5-FU) in the treatment of cancer. A phase II study was initiated to evaluate the effect of a combination of 5-FU/FA/IFN-alpha in patients with advanced pancreatic cancer. Sixty previously untreated patients with advanced adenocarcinoma of the pancreas were treated with 500 mg m-2 FU via an intravenous bolus 1 h after the initiation of a 2 h infusion of 500 mg m-2 FA. Before starting the FA infusion, 6 million units (MU) of IFN-alpha was administered subcutaneously. The treatment was repeated once a week. Of 57 evaluable patients, eight (14%) had a partial response (PR), eight (14%) a minor response (MR) and 28 (49%) no change of disease (NC). Thirteen patients (23%) had progressive disease (PD). The median survival time was 10 months for all patients, 22 months for patients with partial remission and 5 months for patients with progressive disease. Many patients with tumour-related pain whose tumours were affected in terms of PR, MR, NC were free of pain during treatment with this regimen (22/36 patients). The common toxicities observed were fever (56%), nausea (37%) and diarrhoea (33%). These data suggest that biochemical modulation of 5-FU with FA and IFN-alpha has some positive effects in the treatment of pancreatic cancer of moderate toxicity.


British Journal of Cancer (1995) 71, 102-105

© 1995 Stockton Press All rights reserved 0007-0920/95 $9.00

### Treatment of advanced pancreatic cancer with 5-fluorouracil, folinic acid and interferon alpha-2A: results of a phase II trial

H Bernhard[1], E Jäger-Arand[2], G Bernhard[3], M Heike[1], O Klein[2], JF Riemann[4], K-H Meyer zum Büschenfelde[1], W Dippold[1] and A Knuth[2]

[1]*I. Medizinische Klinik und Poliklinik, Johannes Gutenberg-Universität, Langenbeckstraße 1, 55101 Mainz, Germany;* [2]*II. Medizinische Klinik, Krankenhaus Nordwest, Steinbacher Hohl 2-26, 60488 Frankfurt am Main, Germany;* [3]*Institut für Medizinische Statistik und Dokumentation, Johannes Gutenberg-Universität, Langenbeckstraße 1, 55101 Mainz, Germany;* [4]*Medizinische Klinik C, Klinikum der Stadt Ludwigshafen am Rhein, 67063 Ludwigshafen, Germany.*

**Summary** Interferon alpha-2a (IFN-α) and folinic acid (FA) have been shown to modulate the cytotoxic effects of 5-fluorouracil (5-FU) in the treatment of cancer. A phase II study was initiated to evaluate the effect of a combination of 5-FU/FA/IFN-α in patients with advanced pancreatic cancer. Sixty previously untreated patients with advanced adenocarcinoma of the pancreas were treated with 500 mg m$^{-2}$ FU via an intravenous bolus 1 h after the initiation of a 2 h infusion of 500 mg m$^{-2}$ FA. Before starting the FA infusion, 6 million units (MU) of IFN-α was administered subcutaneously. The treatment was repeated once a week. Of 57 evaluable patients, eight (14%) had a partial response (PR), eight (14%) a minor response (MR) and 28 (49%) no change of disease (NC). Thirteen patients (23%) had progressive disease (PD). The median survival time was 10 months for all patients, 22 months for patients with partial remission and 5 months for patients with progressive disease. Many patients with tumour-related pain whose tumours were affected in terms of PR, MR, NC were free of pain during treatment with this regimen (22/36 patients). The common toxicities observed were fever (56%), nausea (37%) and diarrhoea (33%). These data suggest that biochemical modulation of 5-FU with FA and IFN-α has some positive effects in the treatment of pancreatic cancer of moderate toxicity.

**Keywords:** pancreatic carcinoma; 5-fluorouracil; folinic acid; interferon alpha-2a; phase II trial

Pancreatic cancer is often diagnosed when the disease is far advanced. Only a minority of patients can be cured by surgery after early diagnosis. Patients with inoperable pancreatic cancer have a limited survival rate, averaging only 3-4 months (Warshaw *et al.*, 1992; Brennan *et al.*, 1993). The results of chemotherapy for pancreatic carcinoma have generally been disappointing, and only a few agents appear to have a proven therapeutic effect. 5-Fluorouracil is the most evaluated and commonly used single agent. Response rates achieved range from 10 to 20%. Other substances, such as mitomycin, ifosfamide or epirubicine, do not induce greater response rates. Furthermore, combination therapies are not superior to single-agent 5-FU and cause more side-effects (Cullinan *et al.*, 1990; Kelsen *et al.*, 1991; Wils, 1991; Warshaw *et al.*, 1992; Brennan *et al.*, 1993). However, 5-FU alone does not improve survival rates, and to date chemotherapy must be considered experimental in this disease. Attempts to improve the therapeutic efficacy of 5-FU are focused on the principles of 'biomodulation' of the cellular effects of its metabolites. Preclinical studies with biomodulators such as folinic acid or IFN-α have shown an augmentation of the 5-FU cytotoxicity against tumour cells by increasing the 5-FU-induced inhibition of key enzymes of *de novo* DNA synthesis. Folinic acid enhances the 5-FU-induced inhibition of thymidilate synthetase and IFN-α inhibits thymidine kinase (Ernstoff *et al.*, 1989, Grem *et al.*, 1992; Van der Wilt *et al.*, 1992). Considering that some clinical trials on the biomodulation of 5-FU with IFN-α and/or folinic acid in colorectal carcinoma have been successful, similar therapy regimens seem to be worth testing in pancreatic cancer (Petrelli *et al.*, 1987, 1989; Wadler *et al.*, 1989; Bukowski *et al.*, 1991; Piedbois *et al.*, 1992; Sobrero *et al.*, 1992).

We therefore conducted a pilot study to evaluate the toxicity of the combination 5-FU/FA/IFN-α in patients with pancreatic cancer by adding IFN-α to the schedule of 5-FU and FA (Knuth *et al.*, 1992), which had been introduced by Petrelli *et al.* in colorectal cancer (Petrelli *et al.*, 1987, 1989). Because of the promising trends shown there, we initiated this phase II trial to study the efficacy and the toxicity of the double modulation of 5-FU through FA and IFN-α in a larger group of patients.

### Patients and methods

### *Eligibility criteria*

All patients had histologically proven, progressive adenocarcinoma of the pancreas. Inoperability was defined by computerised tomographic (CT) scan and in some borderline cases by additional angiography. All patients had bidimensionally measurable tumour parameters. Patients were followed for 6 weeks prior to treatment to document progressive disease before therapy was initiated. The criteria for tumour progression prior to therapy were not strictly according to the WHO classification which was used for the response criteria because advanced pancreatic carcinoma causes death in a very short period of time, even if the tumour does not grow >25% in cross-sectional area. Patients suffering from tumour-related symptoms (e.g. pain) were classified as having a progressive disease and treatment was started at once. Eligibility requirements included adequate bone marrow (WBC $\geqslant 3.5 \times 10^9$ l$^{-1}$, platelet count $\geqslant 100 \times 10^9$ l$^{-1}$), liver (serum bilirubin $\leqslant 85$ μmol l$^{-1}$) and kidney function (serum creatinine $\leqslant 150$ μmol l$^{-1}$). Furthermore, Karnofsky performance status had to be 60% or greater and estimated life expectancy more than 8 weeks. Patients with CNS metastases, prior external beam radiation therapy of tumour manifestations or concomitant malignancies were ineligible. A formal consent to participate in this protocol was required from all patients.

### *Study design*

The study regimen was designed as follows: 500 mg m$^{-2}$ 5-FU as i.v. bolus 1 h after initiation of a 2 h infusion of 500 mg m$^{-2}$ folinic acid and 6 MU of IFN-α (Roferon,

Correspondence: A Knuth, Chefarzt der II. Medizinichen Klinik, Haematologie/Onkologie, Krankenhaus Nordwest, Steinbacher Hohl 2-26, D-60488 Frankfurt am Main, Germany
Received 8 February 1994; revised 7 August 1994; accepted 15 August 1994

Hoffman-LaRoche, Germany) subcutaneously once a week (Figure 1). Patients were questioned about side-effects by the responsible physician before each weekly dose. Treatment-related toxicities were documented using the grading of the 'World Health Organization' (WHO) (Miller *et al.*, 1981). Patients with stomatitis, diarrhoea or myelosuppression WHO grade 2 during therapy received a 20% reduced dose of 5-FU (400 mg $m^{-2}$). Treatment was postponed when side-effects of WHO grade $\geqslant 2$ occurred. After recovery, the therapy was continued with a reduced 5-FU dosage (400 mg $m^{-2}$). The dose of IFN-α was modified if there were neurological symptoms or worsening of performance status attributed to treatment (3 MU). End points of therapy included complete response to therapy, partial response, minor response and disease stabilisation for more than 2 months, progressive disease or unacceptable toxicity not responding to dose modification. When PR, MR or NC was demonstrated, treatment was continued for additional 8 weeks in order to confirm the maximal inducible response. After the greatest possible response had been achieved, treatment was not maintained. However, when tumour progression reoccurred during treatment pause, therapy was restarted.

*Response criteria*

Patients were evaluated using weekly histories including physical examination and blood counts. Utilisation and dosage of narcotic medications were documented once a week. All patients suffering from tumour-related pain received morphine-based analgesics at the beginning. Pain relief was called 'disappearance of pain' in those patients who did no longer need any kind of analgesics. Radiological examinations and sonographic or CT scans of measurable tumour parameters were performed every 4 weeks. 'Complete response' (CR) was defined as total disappearance of all tumour manifestations initially observed with no evidence of new areas of malignant disease. 'Partial response' (PR) required a greater than 50% reduction in the sum of the products of the longest perpendicular diameters of all measurable indicator lesions. Tumour reduction smaller than 50% for more than 2 months was designated 'minor response' (MR) and tumour stabilisation longer than 2 months as 'no change' (NC). 'Progressive disease' (PD) was defined as a greater than 25% increase in known malignant disease. Development of ascites was also valued as disease progression. Tumour markers CEA and CA19-9 were measured before and routinely every 4 weeks during therapy. Tumour marker regression of more than 20% was defined as 'decrease in tumour markers'. When both tumour markers were elevated, only the parallel reduction in CEA plus CA19-9 was measured as a 'decrease'.

*Statistical analysis*

Survival curves were estimated by lifetable analysis using the Kaplan-Meier method (Kaplan *et al.*, 1958). To compare survival rates, we calculated the median survival time and progression-free survival time. The number of events was described by proportions in per cent and the exact confidence interval of proportions.

**Figure 1** Therapy schedule.

**Results**

*Patients*

A total of 60 consecutive patients were entered into this trial. Fifty-seven patients were evaluable for response; three patients were not evaluable because of 'loss to follow-up'. Nearly all patients (56/57) had a primary tumour or a local relapse, 25/57 patients had additional metastases and 1/57 patients had only metastases without evidence of a local tumour recurrence. Further details of the patient characteristics are described in Table I.

*Response*

Partial responses were observed in 8 of 57 (14%) patients (exact 95% confidence interval, 7-26%; Table II). The response durations in these eight patients were 2-10 months. There were no complete responses to therapy. Eight patients (14%) had a minor response, i.e. a tumour reduction smaller than 50%, and 28 patients (49%) had stable disease. Thirteen patients (23%) experienced a disease progression. In general, metastases of the lung were more sensitive to therapy than liver metastases or primary tumours. In the five patients with primary tumours and additional lung metastases, regression of the lung nodules was observed after 4-8 weeks of therapy, even though the primary tumour shrank at a later time point (one patient), regressed less than the lung nodules (one patient) or was progressive (three patients). Many patients with tumour-related pain (22/36) experienced relief in association with a PR, MR or NC of measurable disease (Table III). A decrease in elevated tumour markers (CEA, CA19-9) was observed in 14/20 patients with pain relief. This indicates that a minimal tumour reduction, which could not be detected by CT, might be sufficient for the reported pain relief. As soon as tumour-related pain disappeared, treatment with morphine-based analgesics was ended. If tumours progressed while treatment was suspended, therapy was restarted. Interestingly, 3/9 patients responded again during the second course of therapy (1/9 PR; 1/9 MR; 1/9 NC).

**Table I** On study patients characteristics (n = 57)

| | |
|---|---|
| Sex (F/M) | 15/42 |
| Age (median, range) | 60 (32-79) |
| KPS (median, range) | 80 (60-100) |
| Site of disease | |
| Pancreas only | 31 |
| Pancreas plus metastases | 25 |
| Metastases only | 1 |

**Table II** Response to therapy (n = 57)

| | |
|---|---|
| Partial response | 8/57 |
| Minor response | 8/57 |
| No change | 28/57 |
| Progressive disease | 13/57 |
| Median progression-free time (months) | |
| All patients | 5 |
| Responding patients (PR) | 9 |
| Median survival time (months) | |
| All patients (range) | 10 |
| Responding patients (PR) | 22 |
| Patients with local disease | 8 |
| Patients with additional metastases | 10 |

**Table III** Tumour-related pain dependent on the treatment success (n = 36)

| | Tumour-related pain | | |
|---|---|---|---|
| | *More* | *Same* | *Disappeared* |
| Partial response (5) | 0/5 | 0/5 | 5/5 |
| Minor response (2) | 0/2 | 0/2 | 2/2 |
| No change (20) | 0/20 | 5/20 | 15/20 |
| Progression (9) | 6/9 | 3/9 | 0/9 |

**Figure 2** Probability of survival after the initiation of therapy in 57 eligible patients.

**Table IV** Maximum toxicity (n = 57)

| | *WHO grade* | | | | |
|---|---|---|---|---|---|
| | *0* | *1* | *2* | *3* | *4* |
| Anaemia | 53 | 2 | 1 | 1 | |
| Thrombocytopenia | 56 | 1 | | | |
| Leucocytopenia | 54 | | 1 | 2 | |
| Fever | 25 | 8 | 21 | 3 | |
| Fatigue | 41 | 10 | 5 | 1 | |
| Nausea | 36 | 12 | 6 | 3 | |
| Diarrhoea | 38 | 8 | 5 | 6 | |
| Stomatitis | 56 | 1 | | | |
| Conjunctivitis | 50 | 5 | 2 | | |
| Skin irritation | 53 | 2 | 2 | | |

Median progression-free interval from the beginning of therapy was 5 months for all patients and 9 months for patients with partial remission. At the time of analysis, 17 of 57 patients (30%) were alive and 40 of 57 patients (70%) had died. The median duration of survival was 10 months for all patients, 22 months for patients achieving a partial remission and 5 months for patients with progressive disease (Table II; Figure 2). There was no significant survival difference between patients with locoregional disease only (8 months) and patients with distant metastases (10 months).

### *Toxicity*

Toxicity data are presented in Table IV for all eligible patients with at least 4 weeks follow-up ($n = 57$). The most prominent toxicity associated with 5-FU was nausea (21/57; 37%), which was suppressed in all patients by antiemetic treatment during the following cycles of therapy. Diarrhoea, observed in 19/57 patients (33%), was a common gastrointestinal side-effect, which disappeared regularly after dose reduction of 5-FU. Stomatitis was a rare side-effect (1/57 patients) and severe myelosuppression did not occur. The most frequently observed side-effects caused by interferon-α were fever (56%) and fatigue (28%). Fatal treatment-related neurological disorders described previously were not observed (Wadler *et al.*, 1989). However, it cannot be formally ruled out that one observed stroke may have been induced by IFN-α. In this and two other patients the treatment had to be interrupted because of intolerable side-effects: one patient experienced worsening of her asthma and a second increased severity of angina pectoris. No treatment-related death was encountered.

### Discussion

A focus in the chemotherapy of advanced pancreatic cancer has been to maximise the efficacy of 5-FU through biomodulation. While biomodulation of 5-FU has mainly been tested in colorectal cancer, there are only a few clinical reports of this approach in the therapy of pancreatic cancer. This clinical trial was initiated to evaluate the effects of the combination '5-FU/FA/IFN-α' in advanced pancreatic cancer. The preliminary results of this triple combination used as first-line therapy are encouraging. In spite of the low overall response rate (14%) and the lack of complete remissions, most of the patients experienced palliation. No evidence of tumour progression in the first 2 months was documented in 77% of the patients (PR, MR, NC). In 22 of the 36 patients who suffered from tumour-related pain, pre-existing pain disappeared during treatment (60%), regardless of the quality of response (PR, MR or NC). We suggest that the reason for treatment-related pain relief–even in patients with stable disease–is a reduction of tumour infiltration into the coeliac plexus, which cannot be detected by CT. Interestingly, in many patients with pain reduction, a decrease in elevated tumour markers (CEA and/or CA19-9) was observed (14/20 patients).

Toxicities were primarily moderate and responded to dose reduction or specific therapy of the side-effects. Therefore treatment could be carried out in an out-patient setting, which generally is considered an advantage in the palliative treatment of cancer. Moreover, the treatment was not continuous. Therapy was suspended when a stable 'response' to therapy – i.e. a partial remission, minor response or tumor stabilisation–was achieved over a period of at least 2 months. If the tumour progressed during a treatment-free interval, therapy was restarted. The overall survival period of 10 months for all patients appears to be superior to reported survival times of 3–6 months with 5-FU monotherapy (Brennan *et al.*, 1989; Cullinan *et al.*, 1990; Wils, 1991).

Other trials using IFN-α or/and folinic acid in advanced pancreatic cancer have shown marginal activity with respect to response rates and survival (Crown *et al.*, 1991; DeCaprio *et al.*, 1991; Pazdur *et al.*, 1992; Scheithauer *et al.*, 1992; Weinerman and McCormick, 1992; Rubin *et al.*, 1992). Scheithauer *et al.* (1992) recently reported an overall survival time of 5.5 months for patients treated with '5-FU/FA/IFN-α' and Weinermann and McCormick (1992) observed an overall survival time of 20 weeks. Whether these contradictory results are due to patient selection or to different treatment schedules remains to be seen.

Patients with pancreatic carcinoma frequently present with a low performance status, signs of malabsorption owing to pancreatic excretory dysfunction, weight loss, abdominal pain, bowel dysmotility and obstructive jaundice. Because of this constellation of symptoms, most patients do not tolerate side-effects of intensive chemotherapy, but require palliative therapy. Hence, criteria for successful therapy must be not only reponse rate and survival, but also treatment-related toxicity and quality of life. The data from our study suggest that combination of 5-FU with FA and IFN-α has moderate side-effects and may alleviate or abolish pre-existing tumour-related pain. In this respect study patients experienced a palliative benefit from this therapy schedule.

The trends observed in this phase II trial are promising, but further clinical trials are necessary to confirm our preliminary results. To date it is still unclear if 5-FU/FA/IFN-α renders any advantage over 5-FU alone or in combination with FA in the treatment of pancreatic cancer. We have started a randomised trial in order to compare the effectiveness of the combination '5-FU/FA/IFN-α' with '5-FU/FA' alone. This phase III trial will evaluate study parameters regarding quality of life besides response rate and survival.

**References**

BRENNAN MF, KINSELLA TJ AND CASPER ES. (1993). Cancer of the Pancreas. In *Cancer, Principles and Practice of Oncology*, De Vita VT, Hellmann S and Rosenberg SA. (eds). pp 849–882. JB Lippincott: Philadelphia.

BUKOWSKI RM, INOSHITA G, YALAVARTHI P, MURTHY S, GIBSON V, BUDD GT, SERGI JS, BAUER L AND PRESTIFILIPPO J. (1991). A phase I trial of 5-fluorouracil, folinic acid and alpha-2a-interferon in patients with metastatic colorectal carcinoma. *Cancer*, **69**, 889–892.

CROWN J, CASPER ES, BOTET J, MURRAY P AND KELSEN DP. (1991). Lack of efficacy of high-dose leucovorin and fluorouracil in patients with advanced pancreatic adenocarcinoma. *J. Clin. Oncol.*, **9**, 1682–1686.

CULLINAN S, MOERTEL CG, WIEAND HS, SCHUTT AJ, KROOK JA, FOLEY JF, NORRIS BD, KARDINAL CG, TSCHETTER LK AND BARLOW JF. (1990). A phase III trial on the therapy of advanced pancreatic carcinoma. *Cancer*, **65**, 2207–2212.

DECAPRIO JA, MAYER RJ, GONIN R AND ARBUCK G. (1991). Fluorouracil and high-dose leucovorin in previously untreated patients with advanced adenocarcinoma of the pancreas: results of a phase II trial. *J. Clin. Oncol.*, **9**, 2128–2133.

ERNSTOFF M, LEMBERSKY BC & KIRKWOOD JM. (1989). Fluorouracil, interferon-alpha, and colon cancer: rational pursuit of synergism between antimetabolites and biologicals. *J. Clin. Oncol.*, **7**, 1764–1765.

GREM JL, CHU E., BOARMAN D, BALIS FM, MURPHY RF, MCATEE N AND ALLEGRA CJ. (1992). Biochemical modulation of fluorouracil with leucovorin and interferon: preclinical and clinical investigations. *Semin. Oncol.*, **19**, 36–44.

KAPLAN EL AND MEIER P. (1958). Nonparametric estimation from incomplete observations. *J. Am. Stat. Assoc.*, **53**, 457–481.

KELSEN D, HUDIS C, NIEDZWIECKI D, DOUGHERTY J, CASPER E, BOTET J, VINCIGUERRA V AND ROSENBLUTH R. (1991). A phase III comparison trial of streptozotocin, mitomycin and 5-fluorouracil with cisplatin, cytosine arabinoside, and caffeine in patients with advanced pancreatic carcinoma. *Cancer*, **68**, 965–969.

KNUTH A, BERNHARD H, KLEIN O AND MEYER ZUM BÜSCHENFELDE, K-H. (1992). Combination fluorouracil, folinic acid, and interferon alpha-2a: an active regimen in advanced pancreatic carcinoma. *Semin. Oncol.*, **19**, 211–214.

MILLER AB, HOOGSTRATEN B, STAQUET M AND WINKLER A. (1981). Reporting results of cancer treatment. *Cancer*, **47**, 207–214.

PAZDUR R, AJANI JJ, ABBRUZZESE JL, BELT RJ, DAKHIL SR, DUBOVSKY D, GRAHAM S, PILAT S, WINN R AND LEVIN B. (1992). Phase II evaluation of fluorouracil and recombinant α-2a interferon in previously untreated patients with pancreatic adenocarcinoma. *Cancer*, **70**, 2073–2076.

PETRELLI N, HERRERA L, RUSTUM Y, BURKE P, CREAVEN P, STULC J, EMRICH LJ AND MITTELMAN A. (1987). A prospective randomized trial of 5-fluorouracil versus 5-fluorouracil and high-dose leucovorin versus 5-fluorouracil and methotrexate in previously untreated patients with advanced colorectal carcinoma. *J. Clin. Oncol.*, **5**, 1559–1565.

PETRELLI N, DOUGLASS HO, HERRERA L, RUSSEL D, STABLEIN DM, BRUCKNER HW, MAYER RJ, SCHINELLA R, GREEN MD, MUGGIA FM AND THE GASTROINTESTINAL TUMOR STUDY GROUP. (1989). The modulation of fluorouracil with leucovorin in metastatic colorectal carcinoma: a prospective randomized phase III trial. *J. Clin. Oncol.*, **7**, 1419–1426.

PIEDBOIS P, BUYSE M., RUSTUM Y, MACHOVER C, ERLICHMAN C, CARLSON RW, VALONE F, LABBIANCA R, DOROSHOW JH AND PETRELLI N. (1992). Modulation of fluorouracil by leucovorin in patients with advanced colorectal cancer: evidence in terms of response rate. *J. Clin. Oncol.*, **10**, 896–903.

RUBIN J, GALLAGHER J, SCHUTT A, KUROSS S AND WIEAND H. (1992). Phase II trials of 5-fluorouracil (5-FU) and leucovorin (LV) in patients with metastatic gastric or pancreatic carcinomas (abstract). *Proc. Am. Soc. Clin. Oncol.*, **11**, 496.

SCHEITHAUER W, PFEFFEL F, KORNEK G, MARCZELL A, WILTSCHKE C AND FUNOVICS J. (1992). A phase II trial of 5-fluorouracil, leucovorin and recombinant alpha-2b-interferon in advanced adenocarcinoma of the pancreas. *Cancer*, **70**, 1864–1866.

SOBRERO A, NOBILE MT, GUGLIELMI A, MORI A, ASCHELE C, BOLLI E, TIXI L, GALLO L, PARODI GC AND BRUZZI P. (1992). Phase II study of 5-fluorouracil plus leucovorin and interferon alpha 2b in advanced colorectal cancer. *Eur. J. Cancer*, **82**, 850–852.

VAN DER WILT CL, PINEDO HM, SMID K, CLOOS J, NOORDHUIS P AND PETERS GJ. (1992). Effect of folinic acid on fluorouracil activity and expression of thymidilate synthase. *Semin. Oncol.*, **19**, 16–25.

WADLER S, SCHWARTZ EL, GOLDMAN M, LYVER A, RADER M, ZIMMERMAN M, ITRI L, WEINBERG V AND WIERNIK PH. (1989). Fluorouracil and recombinant alpha-2a-interferon: an active regimen against advanced colorectal carcinoma. *J. Clin. Oncol.*, **7**, 1769–1775.

WARSHAW AL AND FERNANDEZ-DEL C. (1992). Pancreatic cancer. *N. Engl. J. Med.*, **326**, 455–465.

WEINERMAN B AND McCORMICK R. (1992). A phase 2 survival comparison of patients with pancreatic cancer, treated with 5-fluorouracil and calcium leucovorin compared to matched tumor registry (abstract). *Proc. Am. Soc. Clin. Oncol.*, **11**, 577.

WILS JA. (1991). Chemotherapy in pancreatic cancer: a rational persuit? *Anti-Cancer Drugs*, **2**, 3–10.